# Atrial fibrillation and thromboembolic risk in hypertrophic cardiomyopathy

**DOI:** 10.1186/s44348-025-00057-2

**Published:** 2025-08-25

**Authors:** You-Jung Choi, Neal K. Lakdawala

**Affiliations:** 1 Biomedical Research Institute, Seoul National University Hospital, Seoul, Republic of Korea; 2 Department of Genetics, Harvard Medical School, Boston, MA USA; 3 Department of Cardiovascular Medicine, Brigham and Women’s Hospital, Harvard Medical School, Boston, MA USA

**Keywords:** Ischemic stroke, Arrhythmias, Cardiac, Cardiomyopathies

## Abstract

Atrial fibrillation (AF) is the most common sustained arrhythmia in patients with hypertrophic cardiomyopathy (HCM), conferring a markedly increased risk of thromboembolic events. Conventional risk stratification tools such as the CHA_2_DS_2_-VASc (congestive heart failure, hypertension, age ≥ 75 years [doubled], diabetes mellitus, prior stroke or transient ischemic attack [doubled], vascular disease, age 65–74 years, female sex) score are often insufficient to predict thromboembolic events in patients with HCM and AF, as thromboembolic risk in HCM is driven by disease-specific structural, functional, and prothrombotic substrates. This review synthesizes current evidence on the epidemiology, pathophysiological mechanisms, and clinical impact of AF and thromboembolism in HCM. We discuss variable imaging modalities—including strain echocardiography, cardiac magnetic resonance, and cardiac computed tomography—that offer enhanced characterization of atrial remodeling and thromboembolic risk in patients with HCM. Furthermore, we outline current guideline-based anticoagulation strategies, the evolving role of direct oral anticoagulants, and adjunctive therapies such as left atrial appendage occlusion and catheter ablation. A comprehensive, multidisciplinary approach that incorporates advanced imaging, molecular profiling, and individualized management is ideal to optimize outcomes and reduce stroke burden in patients with HCM and AF.

## Background

Hypertrophic cardiomyopathy (HCM) is the most common inherited cardiomyopathy, characterized by unexplained left ventricular (LV) hypertrophy in the absence of loading conditions, such as hypertension or aortic stenosis. Its hallmark histopathologic features include myocyte disarray, interstitial fibrosis, and small vessel disease [1–3]. The prevalence of HCM in the general population is estimated to be approximately 1 in 200 to 1 in 500 individuals [4, 5]. The clinical presentation of HCM is highly variable, ranging from asymptomatic disease to progressive heart failure and sudden cardiac death. While HCM has been associated with adverse outcomes, including sudden cardiac death, heart failure, and arrhythmic complications, recent advances in early detection, longitudinal surveillance, and risk-guided management have significantly improved prognosis [6–8].

Atrial fibrillation (AF) represents the most common sustained arrhythmia in HCM, with a prevalence significantly higher than observed in the general population [9, 10]. This carries significant clinical implications, as the presence of AF in HCM is associated with a markedly increased risk of thromboembolism—often exceeding predictions from conventional risk stratification tools [9–15]. The heightened thromboembolic risk is attributed to the complex interplay among structural abnormalities, electrical remodeling, and the hypercoagulable state of HCM [16–19]. Current clinical guidelines recommend anticoagulation for all patients with HCM and AF, regardless of traditional stroke risk factors, unless contraindicated [20, 21].

This review provides a comprehensive overview of the epidemiology, underlying pathophysiologic mechanisms, and evolving strategies for risk stratification and management of AF and thromboembolism in HCM, with particular emphasis on the crucial role of multimodality imaging in clinical decision-making.

## Epidemiology of AF and thromboembolism in HCM

AF is the most prevalent sustained arrhythmia in patients with HCM, occurring at a substantially higher rate than in the general population. The reported prevalence of AF in HCM ranges from 20 to 30% [9–11, 13, 14], with longitudinal studies estimating an annual incidence of new-onset AF of approximately 2%to 4% [9, 12, 22]. In patients with HCM over the age of 70 years, the lifetime prevalence of AF may approach 40% to 50% [10]. Overall, nearly one-third of individuals with HCM experience at least one episode of AF [12]. Among these, about two-thirds of AF episodes are paroxysmal, terminating spontaneously or with intervention within 7 days, while one-third are persistent and may progress to permanent AF [10, 23].

AF in HCM is associated with disproportionately high risk of thromboembolic events, even in patients with low CHA_2_DS_2_-VASc (congestive heart failure, hypertension, age ≥ 75 years [doubled], diabetes mellitus, prior stroke or transient ischemic attack [doubled], vascular disease, age 65–74 years, female sex) scores. The annual incidence of thromboembolic events in patients with both HCM and AF ranges from 3.8% to 7.6%, substantially exceeding the 1% to 2% yearly risk observed in the non-HCM AF population with similar CHA_2_DS_2_-VASc scores [9, 13]. In particular, cohort studies have reported annual stroke rates exceeding 3% in anticoagulation-naive patients with HCM and AF, underscoring the inadequacy of conventional risk stratification tools in this population [15]. Notably, even a single isolated paroxysmal episode of AF in HCM is associated with a significant risk of thromboembolism, in contrast to the general AF population, where sustained or permanent AF more strongly predicts adverse events [10, 14].

In addition to thromboembolic risk, AF has important prognostic implications in patients with HCM. AF is independently associated with increased mortality, with annual HCM-related mortality rates two to three times higher in patients with AF compared to those in sinus rhythm [12, 22, 24]. This excess mortality is largely attributed to both progressive heart failure and thromboembolic events. Importantly, the onset and progression of AF is not a simple phenomenon, but an important inflection point in the trajectory of the disease, often accompanied by acute decompensated heart failure, increased hospitalization, and increased risk of death [25–28].

## Pathophysiological mechanism of AF and thromboembolism in HCM

### Structural and functional substrate for AF

Left atrial (LA) remodeling stands as the central pathophysiological mechanism underlying AF in patients with HCM [29]. This remodeling process arises in response to the chronic pressure and volume overload, characteristics of HCM, resulting primarily from impaired myocardial relaxation and increased chamber stiffness. These hemodynamic abnormalities lead to sustained elevation in LV filling pressure, resulting in progressive LA dilation, increased wall stress, and the development of interstitial fibrosis [30–32].

Consistent with this remodeling, histopathological analyses of atrial tissue from patients with HCM have revealed extensive interstitial fibrosis and myocyte disarray, which collectively establish a vulnerable arrhythmogenic substrate capable of sustaining reentry circuits and ectopic foci that can initiate and maintain AF [30, 33]. Cardiac magnetic resonance imaging (MRI) also frequently demonstrates late gadolinium enhancement (LGE), a noninvasive marker of myocardial fibrosis, in both ventricles and atrial myocardium. The presence and extent of LGE in both LA and LV have been shown to correlate strongly with the risk of AF burden and recurrence risk, reflecting the underlying fibrotic remodeling [34–36]. In parallel, electrical remodeling occurs alongside these structural alterations, characterized by shortened atrial action potential duration, altered ion channel expression, and reduced conduction velocity [37–39]. Taken together, these structural and functional alterations create a permissive substrate for both the initiation and maintenance of AF in the setting of HCM.

Among the hemodynamic abnormalities in HCM, LV outflow tract (LVOT) obstruction is particularly influential in promoting LA remodeling and AF [40]. Present in approximately 40% to 60% of patients with HCM, either at rest or with provocation, LVOT obstruction is a major contributor to impairing LA mechanics [41, 42]. A key mechanism linking LVOT obstruction to LA remodeling is mitral regurgitation (MR), which occurs in 60% to 70% of patients with HCM, and is often secondary to systolic anterior motion of the mitral valve or intrinsic valve abnormalities [43–45]. The severity of MR has been positively correlated with both LA structural and functional impairment, as well as increased risk of AF in patients with HCM [43, 46].

Myocardial ischemia, which can occur in HCM due to microvascular dysfunction and supply–demand mismatch, may further contribute to atrial electrical instability, thereby serving as an additional trigger for AF in predisposed individuals through multiple mechanisms [47–49]. These mechanisms include worsening LV diastolic function, elevated LV filling pressure, increased LA stretch, atrial ischemia resulting from microcirculatory dysfunction, atrial fibrosis, and enhanced arrhythmogenicity. However, these proposed pathways remain largely speculative to date.

Once AF develops, it initiates a vicious cycle by eliminating coordinated atrial contraction, increasing atrial pressure, and promoting further atrial dilation and dysfunction [50]. This perpetuates the arrhythmogenic substrate and contributes to persistent arrhythmia maintenance [31]. Notably, AF also promotes blood stasis, particularly within the LA appendage (LAA), which is the most common site of thrombus formation. Even in sinus rhythm, LAA dysfunction is not uncommon in HCM and is markedly worsened by AF, thereby increasing the risk of thromboembolism [10, 19, 51].

### Molecular and genetic considerations

Several sarcomeric gene variants—most frequently in *MYH7*, which encodes β-myosin heavy chain, and *MYBPC3*, which encodes myosin-binding protein C—are well-established genetic drivers of HCM. These pathogenic variants are frequently associated with more pronounced myocardial disarray, an earlier onset of disease, and a greater degree of interstitial fibrosis, compared to noncarriers [17, 52]. Recent advances in genotype–phenotype correlating studies have begun to illuminate how specific variants may predispose individuals to a more arrhythmogenic atrial substrate [53]. For example, pathogenic *MYH7* variants have been linked to a higher incidence of new-onset AF in patients with HCM, with a hazard ratio of 1.72 (95% confidence interval, 1.27–2.33) compared to *MYBPC3* variants, based on data from the multinational registry of 1,040 genotype-positive, AF-free adult HCM patients followed for a mean of 7.2 years [54]. Patients with *MYH7* variants were more likely to present at a younger age and exhibit phenotypic features such as LA hypertrophy, dilatation, and extensive fibrosis—structural substrates that facilitate arrhythmia initiation and maintenance [54, 55]. In contrast, patients with *MYBPC3* variants tended to be older at diagnosis, were less frequently probands, and more often underwent septal reduction therapy before developing AF, with a relatively attenuated association with atrial arrhythmias [54]. These observations underscore the importance of integrating genetic information into clinical risk stratification frameworks, as it may offer valuable insights into individualized arrhythmia risk and thromboembolic potential.

Beyond sarcomeric mutation, nonsarcomeric genetic factors have also been implicated in modulating AF susceptibility in patients with HCM. Genome-wide association studies have identified common susceptibility loci for AF, most notably *PITX2* and *ZFHX3*, and *KCNN3,* which appear to overlap with loci implicated in familial AF [56]. These nonsarcomeric loci may contribute to a heritable predisposition for atrial arrhythmias and thromboembolic events in patients with HCM, independent of the degree of structural remodeling.

### Mechanisms underlying thromboembolic risk in HCM

The development of AF in patients with HCM is associated with a markedly increased risk of thromboembolic events, often disproportionate to conventional risk stratification metrics such as CHA_2_DS_2_-VASc scores [12, 14, 57, 58]. This heightened thrombotic risk in HCM reflects a distinct pathophysiologic profile.

A major contributor to thromboembolism in HCM is LA stasis, driven by a combination of atrial enlargement, impaired contractile function, and altered geometry that creates regions of blood stagnation, particularly in the LAA [19, 51]. Transesophageal echocardiography (TEE) has revealed that patients with HCM often exhibit reduced LAA emptying velocities and more complex morphologies, both independently associated with thrombus formation [51].

In addition to structural abnormalities, endocardial dysfunction plays a key role in thrombogenesis in HCM. Patients with HCM exhibit systemic evidence of endothelial dysfunction, as reflected by upregulated circulating levels of soluble thrombomodulin and tissue factor pathway inhibitor, including thrombomodulin and endothelial protein C [59]. Notably, these abnormalities were more pronounced in patients with LVOT obstruction [59]. Even in the absence of AF, LVOT obstruction is independently associated with enhanced thrombin generation and platelet activity in patients with HCM [60]. These alterations create endothelial activation and disruption of anticoagulant balance, potentially contributing to a prothrombotic state.

Furthermore, patients with HCM demonstrate elevated markers of thrombin generation, including plasma levels of fibrinopeptide A and thrombin-antithrombin III complex, compared to healthy controls, indicating activation of the coagulation system [18]. Importantly, these markers showed a positive correlation with LA diameter, implicating LA dilation as a key driver of coagulation activation in HCM.

## Risk assessment for AF and thromboembolism in HCM

### Risk factors for AF in HCM

Multiple clinical and demographic factors have been identified that predict AF development in patients with HCM. Left atrial enlargement represents the most robust and consistently reported risk factor, with numerous studies demonstrating that LA diameter exceeding 40 to 45 mm is associated with a threefold to fourfold increased risk of new-onset AF [9, 12, 24, 61]. Reflecting this evidence, the European Society of Cardiology (ESC) guidelines recommend 48-h ambulatory electrocardiographic monitoring every 6 to 12 months for AF detection in patients with HCM who are in sinus rhythm and have an LA diameter > 45 mm (class IIa recommendation) [62]. Beyond simple diameter measurements, volumetric assessment of LA provides superior prognostic information. An LA volume index greater than 34 mL/m^2^ has been reported to predict new-onset AF with a sensitivity of 82% and a specificity of 73% in patients with HCM [63]. In contrast, a lower LA volume of < 37 mL/m^2^ appears to strongly predict the absence of AF, with a reported negative predicted value of 92% [61].

Age remains a significant risk factor, with each additional decade of life approximately doubling the risk for AF in patients with HCM [12, 31, 64]. Specifically, Olivotto et al. [12] reported that an age at diagnosis greater than 50 years was associated with a twofold increase in the risk of developing AF. Additionally, symptomatic status correlates with AF risk, with studies demonstrating that patients with HCM in New York Heart Association (NYHA) functional class III–IV have approximately threefold to fourfold higher risk of developing AF compared to those with mild or no symptoms [12, 31, 65].

### Risk factors for thromboembolism in HCM

AF is a well-established risk factor for thromboembolic events in patients with HCM. Nevertheless, over half of thromboembolic events occur in the absence of previously documented AF, indicating the involvement of other contributing factors. This is particularly notable among patients aged 65 years of age or older and those with chronic heart failure, who exhibit a 2.7- and 1.8-fold higher risk, respectively [66, 67].

Morphological abnormalities also contribute significantly to thromboembolic risk in HCM. LA enlargement, which is routinely assessed via echocardiography in clinical practice, is not only associated with AF, but also an independent predictor of thromboembolic events in patients with HCM [24, 57]. Moreover, LV morphological features, including apical ballooning, have likewise been associated with a higher prevalence of thrombus formation in patients with HCM [68, 69].

Several clinical comorbidities have also been associated with increased thromboembolic risk. These include a prior history of thromboembolic event and atherosclerotic cardiovascular disease (e.g., myocardial infarction, complex aortic plaque, and peripheral arterial disease), and the presence of NYHA functional class symptoms has also been associated with an elevated risk of thromboembolic events in patients with HCM [57].

Additionally, structural and biochemical markers also contribute to thromboembolic risk in patients with HCM. These include echocardiographic parameters (e.g., LA diameter, maximal wall thickness, and LVOT gradient) [57], cardiac MRI-based assessment of myocardial fibrosis (e.g., LGE > 14.4%) [16], and circulating biomarkers of atrial remodeling (e.g., N-terminal pro–B-type natriuretic peptide and troponins) [70–74]. Among circulating biomarkers, elevated natriuretic peptides reflect elevated filling pressures and the neurohormonal activation that contributes to thrombogenesis. Similarly, high-sensitivity troponin elevation may indicate ongoing myocardial injury and greater hemodynamic compromise, identifying patients with more advanced disease and heightened stroke risk [74, 75].

## Imaging modalities for risk assessment

Imaging modalities for risk assessment are summarized in Table 1.

**Table 1:** Comparative summary of imaging modalities for risk stratification in patients with HCM and AF

Modality	Key feature	Identified risk marker	Clinical application
TTE	LA diameter and volume MR severity and LVOT obstruction LA strain by speckle-tracking echocardiography	LA diameter > 45 mm → ↑ AF risk (threefold–fourfold), indication for 48-h ambulatory ECG monitoring every 6–12 months LA volume index > 34 mL/m^2^ → predicts new-onset AF with sensitivity of 82% and specificity of 73% LA volume index < 37 mL/m^2^ → NPV 92% for absence of AF LA strain < 23% → new-onset AF LA strain < 17% → thromboembolism in AF	Initial screening and routine monitoring LA structural and functional remodeling
TEE	LAA morphology and thrombus LAA flow velocities	LAA emptying velocity < 20 cm/sec → ↑ thrombus, SEC, embolic risk	Preprocedural assessment for cardioversion/ablation or LAA occlusion Identification of LAA thrombus
Cardiac MRI	Gold-standard for myocardial fibrosis assessment (LGE) LA volume and function	LGE > 14.4% → ↑ thromboembolism 10% ↑ LGE → approximately 1.8-fold ↑ AF risk LA strain reduction → ↑ AF/thromboembolism risk Apical aneurysm detection → ↑ thromboembolic risk	Fibrosis-guided AF/thromboembolism risk stratification Evaluation of LA function and strain
Cardiac CT	High-resolution 3D visualization of LAA anatomy Delayed contrast-enhanced imaging for thrombus LAA emptying fraction (not yet widely adopted)	High diagnostic accuracy (> 99%) for LAA thrombus LAA ejection fraction < 40% → ↑ thromboembolism risk	Noninvasive thrombus and anatomical evaluation of LAA (alternative to TEE) Planning for LAA occlusion

*AF* atrial fibrillation, *CT* computed tomography, *ECG* electrocardiogram, *HCM* hypertrophic cardiomyopathy, *LA* left atrium, *LAA*, left atrial appendage, *LGE* late gadolinium enhancement, *LVOT* left ventricular outflow tract, *MR* mitral regurgitation, *MRI* magnetic resonance imaging, *NPV* negative predictive value, *SEC* spontaneous echo contrast, *TEE* transesophageal echocardiography, *TTE*, transthoracic echocardiography

### Echocardiography

Multimodality imaging plays a pivotal role in the assessment of AF and thromboembolic risk in patients with HCM. Echocardiography remains the cornerstone imaging modality in routine clinical practice for diagnosis and risk stratification of HCM, providing critical insights on LA morphologies, LAA assessment, LVOT obstruction, MR severity, and diastolic dysfunction. Transthoracic echocardiography enables accurate measurement of LA diameter and volume, both of which are established predictors of AF and thromboembolism in HCM [24, 61, 63, 76].

Advanced echocardiographic techniques, such as speckle-tracking echocardiography, provide a functional assessment of LA mechanics. Although not yet widely adopted in all clinical settings, LA strain measurement has demonstrated predictive value for incident AF and thromboembolic risk [77, 78]. The reduction in LA reservoir strain is closely linked to LV diastolic dysfunction. Impaired LV relaxation and elevated LV filling pressures increase LA afterload and wall stress, which in turn promote LA fibrosis and impaired compliance. In a cohort of 414 patients with HCM, a novel LA strain-based classification effectively stratified LV diastolic dysfunction [79]. Thus, LA strain reflects both intrinsic atrial dysfunction and the downstream effects of LV diastolic impairment, underscoring its value as an early marker of AF and thromboembolic risk in HCM. Notably, in patients with established AF, reduced LA reservoir strain (< 23% in predicting new-onset AF and < 17% in predicting thromboembolic events) can identify atrial dysfunction even before atrial enlargement becomes apparent [61, 80]. In addition, reduced LA reservoir strain is the best predictor of reduced LAA emptying velocity and LAA thrombus [81].

TEE, though more invasive, is commonly used in practice to evaluate LAA morphology and thrombus [82], particularly in patients undergoing cardioversion or ablation [83]. Specifically, pulsed-wave Doppler–derived LAA emptying velocities < 20 cm/sec indicate an elevated risk of thrombus and spontaneous echocardiographic contrast [84].

### Cardiac MRI

Cardiac MRI has emerged as a key tool for the comprehensive evaluation of patients with HCM, with its ability to characterize myocardial tissue being one of its most valuable strengths. Myocardial fibrosis detected by LGE has been shown to correlate independently with the risk of AF and thromboembolic events in patients with HCM [16, 64]. Notably, an increase in the extent of LGE by 10% has been associated with an approximately 1.8-fold rise in AF risk [64]. In addition, an LGE extent exceeding 14.4% has been identified as an independent predictor for thromboembolic events in patients with HCM [16]. While LGE assessment has traditionally focused on ventricular myocardium, recent studies have highlighted the utility of cardiac MRI in detecting atrial LGE, which correlates with atrial myopathy and AF susceptibility [36, 85]. However, LGE quantification may suffer from limited reproducibility across centers due to variability in image acquisition protocols and post-processing techniques [86]. This limits the generalizability of its prognostic value. Therefore, further efforts to standardize imaging protocols and quantification methods are necessary to improve consistency and facilitate broader clinical implementation.

In addition, cardiac MRI provides a gold-standard assessment of LA volumes and function [87], with feature-tracking techniques allowing detailed strain analysis that has shown strong associations with AF development and thromboembolic events in patients with HCM [29, 88]. Cardiac MRI may be additionally useful for identifying an apical thrombus in patients with HCM and apical aneurysm ballooning [89].

### Cardiac computed tomography

Cardiac computed tomography (CT) is not routinely used for risk stratification in HCM but may provide clinically useful information in selected cases, particularly when echocardiographic images are suboptimal or when planning LAA occlusion procedures. Although evidence in HCM is limited, contrast-enhanced cardiac CT has demonstrated excellent sensitivity and specificity (> 99%) for LAA thrombus detection in broader AF populations, in a recent meta-analysis [90]. In addition, CT offers detailed 3D characterization of LAA anatomy, including volume, number of lobes, and orifice dimensions, which is particularly valuable when planning LAA occlusion procedures [91]. In research and specialized settings, CT-derived LAA emptying fraction (< 40%) [92] and LA strain measurement have shown potential for thromboembolic risk assessment, though their use remains investigational in HCM [93].

## Prevention and management strategies

### Anticoagulation approaches

Given the high incidence of stroke in patients with HCM and AF, current guidelines from major cardiovascular societies warrant a lower threshold for anticoagulation than in the general AF population. The 2024 American Heart Association/American College of Cardiology (AHA/ACC) guidelines for HCM recommend anticoagulation for all patients with HCM and persistent/paroxysmal AF, irrespective of CHA_2_DS_2_-VASc score (class I recommendation) [21]. Similarly, the ESC guidelines provide a class IIa recommendation, supporting anticoagulation for all patients with paroxysmal, persistent, or permanent AF [62]. Importantly, lifelong therapy with oral anticoagulants is advised, even after restoration of sinus rhythm, due to the persistent risk of thromboembolism in the high-risk population.

Historically, vitamin K antagonists (e.g., warfarin) were the mainstay of anticoagulation. However, accumulating observational evidence supports the use of direct oral anticoagulants (DOACs), including factor Xa inhibitors and direct thrombin inhibitors, demonstrating—or in some outcomes, superior—safety profiles regarding intracranial bleeding, hemorrhagic stroke, and major bleeding in patients with HCM and AF [94–97]. There are practical benefits to using DOACs, such as fixed dosing, fewer dietary and drug interactions, and no requirement for routine international normalized ratio monitoring, thereby enhancing convenience and adherence. Standard dosing regimens are appropriate for most patients, with adjustments guided by existing AF criteria based on renal function, age, and body weight. However, in patients with HCM, there are currently no head-to-head randomized controlled trials (RCTs) or guideline-endorsed recommendations favoring one DOAC over another, such as apixaban, rivaroxaban, or edoxaban, leaving the optimal agent undefined. In this context, shared decision-making remains essential, considering individual patient characteristics, comorbidities, bleeding risk, and clinical experience.

### Rhythm control strategies

Restoration and maintenance of sinus rhythm in patients with HCM and AF reduce thromboembolic risk by improving atrial mechanical function and reducing blood stasis [98], as supported by strain imaging studies demonstrating LA reverse remodeling with a concomitant improvement in LA strain after rhythm control [99].

Pharmacological rhythm control in patients with HCM is challenging. Amiodarone is most effective for maintaining sinus rhythm in this population [100, 101], but its long-term toxicity limits its use, especially in younger patients [102]. Sotalol offers moderate efficacy and a more favorable safety profile [103, 104]. In a retrospective study of 98 patients (130 drug treatments) with HCM and symptomatic AF, the probability of remaining on sotalol was 74% at 1 year and 50% at 3 years, with only 2% discontinuing due to side effects and no serious adverse events reported; however, clinical experience in HCM remains limited [104]. Disopyramide is particularly useful in obstructive HCM for symptom relief and, in select cases, rhythm control due to its negative inotropic effects. While randomized trials are lacking, observational data support its medium-term efficacy and safety. In a study of 92 patients, 67% of patients remained on therapy after 7.2 years, with 46% showing resolution of LVOT obstruction [105]. Serious adverse events were rare. However, use remains limited by anticholinergic side effects, the need for inpatient initiation in some cases, and limited availability [106]. Class IC drugs (e.g., flecainide, propafenone) are generally avoided due to their proarrhythmic potential in the presence of structural heart disease. Thus, in the context of HCM, their use should be approached with caution and is typically reserved for selected patients, preferably those with an implantable cardioverter-defibrillator [107].

Catheter ablation is a reasonable option for patients with symptomatic AF who are refractory to medical therapy or prefer a rhythm control strategy [108–110]. However, compared to individuals without structural heart disease, patients with HCM exhibit significantly higher recurrence rates and tend to show greater fibrotic remodeling of the LA and less favorable LA reverse remodeling following catheter ablation [17, 111–113]. Although procedure-related complications are uncommon, patients with HCM tend to have longer hospital stays and higher readmission rates, often due to AF-related symptoms or heart failure [112, 114].

On the other hand, in patients with HCM undergoing cardiac surgery (e.g., myectomy), concomitant surgical AF ablation (Cox-Maze or modified procedures) along with LAA exclusion should be considered. In experienced centers, these combined approaches have achieved freedom from recurrent AF in over 70% at 5 years [115]. Notably, 1-year freedom from AF recurrence is reported to be approximately 44% after catheter ablation, compared to 75% following surgical Maze procedures [10]. In addition, advanced ablation technologies such as pulse field ablation have demonstrated improved safety and efficacy profiles compared to conventional thermal ablation [116].

### LAA occlusion

LAA occlusion represents an alternative strategy for stroke prevention in selected patients with HCM and AF [117, 118]. However, since no RCTs have specifically evaluated LAA occlusion in the HCM population, decisions should therefore be individualized based on a comprehensive assessment of stroke and bleeding risk. Real-world data involving 364 patients with HCM and AF treated with LAA occlusion do not support its use as the primary stroke prevention strategy in patients eligible for long-term oral anticoagulation [118]. In this cohort, patients with HCM and AF undergoing LAA occlusion experienced a higher rate of ischemic stroke (13% vs 8%) and systemic embolism (14% vs 9%) compared with propensity-matched patients receiving oral anticoagulation. In contrast, another large real-world study including 71,980 patients with HCM and AF demonstrated that both surgical and transcatheter LAA occlusion were associated with a lower risk of hemorrhagic stroke and major bleeding compared to those without LAA occlusion, although no significant difference was observed in ischemic stroke [119]. These findings suggest that while LAA occlusion may offer bleeding-related benefits, its role in ischemic stroke prevention, particularly among patients with HCM, remains uncertain.

The 2020 European Heart Rhythm Association/European Association of Percutaneous Cardiovascular Interventions Expert Consensus Statement supports percutaneous LAA occlusion in patients with a high thromboembolic risk who have contraindications to long-term anticoagulation [120]. Similarly, the 2019 AHA/ACC Focused Update assigns a class IIb indication for LAA occlusion in such populations [121]. Although large, prospective RCTs have demonstrated the efficacy of LAA occlusion for stroke prevention, as well as reductions in bleeding risk and cardiovascular mortality in patients with nonvalvular AF, these trials have not included individuals with HCM [122, 123]. Consequently, LAA occlusion may be considered in patients with HCM and AF, who have absolute contraindications to anticoagulation, experience recurrent bleeding despite optimized anticoagulation, or have poor anticoagulation control.

### Emerging role of myosin inhibitor

Recently, mavacamten, a selective cardiac myosin inhibitor, has emerged as a novel therapeutic option for patients with obstructive HCM [124]. In addition to its established benefits in reducing LVOT gradients, improving symptoms, and enhancing exercise capacity, preliminary data suggest that mavacamten may also exert favorable effects on LA hemodynamics [125, 126]. By reducing LV diastolic pressure and improving LA compliance, mavacamten may indirectly mitigate AF burden and support LA reverse remodeling. Ongoing studies are exploring its potential role in modulating atrial arrhythmia susceptibility and long-term stroke risk reduction.

### Prediction models for AF and thromboembolism in HCM

While sudden cardiac death risk stratification has first been the focus of HCM management, increasing attention is now directed toward predicting AF and thromboembolic events, which significantly impact quality of life and long-term outcomes.

The HCM-AF score is an externally validated risk prediction model in a cohort of 1,900 patients with HCM to estimate 2-and 5-year risk of new-onset AF [65]. It incorporates age (+ 3 points per decade), LA dimension (+ 2 points per 6-mm increase), age at HCM diagnosis (–2 points per decade), and heart failure symptoms (+ 3 points if present), stratifying patients into risk categories of low (< 1.0% per year), intermediate (1.0%–2.0% per year), and high (> 2.0% per year). In external validation in a separate cohort, high-risk patients had a 2.7% per year AF incidence versus 0.2% per year in low-risk patients. The model demonstrated good discrimination (C-statistic, 0.70 in the development cohort and 0.68 in the validation cohorts) and outperformed LA dimension alone and non-HCM-specific risk models.

More recently, machine learning models integrating multiple clinical, imaging, genetic, and biomarker data have shown superior predictive accuracy [127]. In a prospective multicenter cohort of 1,069 HCM patients without prior AF, a machine learning model achieved an area under the curve of 0.84, with a sensitivity of 82% and specificity of 76%, respectively, significantly outperforming the HCM-AF score [127].

For thromboembolic events, the R-CHA_2_DS_2_-VASc score modifies the traditional CHA_2_DS_2_-VASc score by integrating HCM-specific variables [128]. In a study of 446 patients with HCM, it demonstrated strong discrimination (C-statistic, 0.77), with thromboembolism rates rising from 0.84 per 100 person-years in the low-risk group (score ≤ 2) to 17.54 per 100 person-years in the high-risk group (score ≥ 8). The HCM Risk-CVA score, though incorporating important clinical variables such as LA size, AF status, and vascular disease, is limited by its complexity [57]. Alternatively, the French HCM score, derived from a large national cohort (n = 32,206), offers a simplified approach based on age, heart failure, AF, prior stroke, smoking, and nutrition status, achieving comparable performance (C-statistic, 0.65–0.70) without imaging data [13].

## Conclusions

AF represents a pivotal complication in HCM, associated with increased morbidity and mortality, primarily driven by heightened thromboembolic risk. Its pathophysiology in HCM is distinct, involving structural remodeling, atrial dysfunction, and prothrombotic states, necessitating tailored risk stratification and management strategies. DOACs are preferred for stroke prevention in patients with HCM and AF, and rhythm control strategies such as advanced catheter ablation and surgical Maze procedures may be effective in selected patients. LAA occlusion may offer an alternative in those with contraindications to anticoagulation, though further validation is needed. Recent advances, including HCM-specific prediction models, machine learning approaches, and multimodality imaging, have improved risk assessment for AF and thromboembolism. Future research should prioritize prospective studies focused on HCM-specific anticoagulation and rhythm control strategies, with the goal of enabling individualized, evidence-based care to improve long-term outcomes.

## Data Availability

No datasets were generated or analysed during the current study.

## Abbreviations

ACC: American College of Cardiology
AHA: American Heart Association
AF: Atrial fibrillation
CHA_2_DS_2_-VASc: Congestive heart failure, hypertension, age ≥ 75 years (doubled), diabetes mellitus, prior stroke or transient ischemic attack (doubled), vascular disease, age 65–74 years, female sex
CT: Computed tomography
DOAC: Direct oral anticoagulant
ESC: European Society of Cardiology
HCM: Hypertrophic cardiomyopathy
LA: Left atrial
LAA: Left atrial appendage
LGE: Late gadolinium enhancement
LV: Left ventricular
LVOT: Left ventricular outflow tract
MR: Mitral regurgitation
MRI: Magnetic resonance imaging
NYHA: New York Heart Association
RCT: Randomized controlled trials
TEE: Transesophageal echocardiography

## References

[1] St John Sutton MG, Lie JT, Anderson KR, O’Brien PC, Frye RL. Histopathological specificity of hypertrophic obstructive cardiomyopathy: myocardial fibre disarray and myocardial fibrosis. Br Heart J. 1980;44:433–43. 7191711 10.1136/hrt.44.4.433 PMC482424

[2] Teare D. Asymmetrical hypertrophy of the heart in young adults. Br Heart J. 1958;20:1–8. 13499764 10.1136/hrt.20.1.1 PMC492780

[3] Maron BJ, Epstein SE, Roberts WC. Hypertrophic cardiomyopathy and transmural myocardial infarction without significant atherosclerosis of the extramural coronary arteries. Am J Cardiol. 1979;43:1086–102. 571670 10.1016/0002-9149(79)90139-5

[4] Semsarian C, Ingles J, Maron MS, Maron BJ. New perspectives on the prevalence of hypertrophic cardiomyopathy. J Am Coll Cardiol. 2015;65:1249–54. 25814232 10.1016/j.jacc.2015.01.019

[5] Maron BJ, Gardin JM, Flack JM, Gidding SS, Kurosaki TT, Bild DE. Prevalence of hypertrophic cardiomyopathy in a general population of young adults: echocardiographic analysis of 4111 subjects in the CARDIA study. Coronary artery risk development in (young) adults. Circulation. 1995;92:785–9. 7641357 10.1161/01.cir.92.4.785

[6] Maron BJ, Rowin EJ, Casey SA, Link MS, Lesser JR, Chan RH, et al. Hypertrophic cardiomyopathy in adulthood associated with low cardiovascular mortality with contemporary management strategies. J Am Coll Cardiol. 2015;65:1915–28. 25953744 10.1016/j.jacc.2015.02.061

[7] Sugiura K, Kubo T, Ochi Y, Miyagawa K, Baba Y, Noguchi T, et al. Very long-term prognosis in patients with hypertrophic cardiomyopathy: a longitudinal study with a period of 20 years. ESC Heart Fail. 2022;9:2618–25. 35652322 10.1002/ehf2.13983 PMC9288772

[8] Lee HJ, Kim J, Chang SA, Kim YJ, Kim HK, Lee SC. Major clinical issues in hypertrophic cardiomyopathy. Korean Circ J. 2022;52:563–75. 35929051 10.4070/kcj.2022.0159 PMC9353251

[9] Guttmann OP, Rahman MS, O’Mahony C, Anastasakis A, Elliott PM. Atrial fibrillation and thromboembolism in patients with hypertrophic cardiomyopathy: systematic review. Heart. 2014;100:465–72. 24014282 10.1136/heartjnl-2013-304276

[10] Rowin EJ, Hausvater A, Link MS, Abt P, Gionfriddo W, Wang W, et al. Clinical profile and consequences of atrial fibrillation in hypertrophic cardiomyopathy. Circulation. 2017;136:2420–36. 28916640 10.1161/CIRCULATIONAHA.117.029267

[11] Maron BJ, Olivotto I, Bellone P, Conte MR, Cecchi F, Flygenring BP, et al. Clinical profile of stroke in 900 patients with hypertrophic cardiomyopathy. J Am Coll Cardiol. 2002;39:301–7. 11788223 10.1016/s0735-1097(01)01727-2

[12] Olivotto I, Cecchi F, Casey SA, Dolara A, Traverse JH, Maron BJ. Impact of atrial fibrillation on the clinical course of hypertrophic cardiomyopathy. Circulation. 2001;104:2517–24. 11714644 10.1161/hc4601.097997

[13] Fauchier L, Bisson A, Bodin A, Herbert J, Spiesser P, Pierre B, et al. Ischemic stroke in patients with hypertrophic cardiomyopathy according to presence or absence of atrial fibrillation. Stroke. 2022;53:497–504. 34601900 10.1161/STROKEAHA.121.034213

[14] Choi YJ, Choi EK, Han KD, Jung JH, Park J, Lee E, et al. Temporal trends of the prevalence and incidence of atrial fibrillation and stroke among Asian patients with hypertrophic cardiomyopathy: a nationwide population-based study. Int J Cardiol. 2018;273:130–5. 30150122 10.1016/j.ijcard.2018.08.038

[15] Jung H, Yang PS, Sung JH, Jang E, Yu HT, Kim TH, et al. Hypertrophic cardiomyopathy in patients with atrial fibrillation: prevalence and associated stroke risks in a nationwide cohort study. Thromb Haemost. 2019;119:285–93. 30602200 10.1055/s-0038-1676818

[16] Hohneck A, Overhoff D, Doesch C, Sandberg R, Rudic B, Tueluemen E, et al. Extent of late gadolinium enhancement predicts thromboembolic events in patients with hypertrophic cardiomyopathy. Circ J. 2020;84:754–62. 32238665 10.1253/circj.CJ-19-0936

[17] Haq IU, Akhiyat N, Al-Shakarchi N, Siontis KC, Mulpuru SK, Sugrue A, et al. Atrial fibrillation substrate and catheter ablation outcomes in MYBPC3- and MYH7-mediated hypertrophic cardiomyopathy. JACC: Clinical Electrophysiology. 2024;10:1380–91. 38819352 10.1016/j.jacep.2024.03.026

[18] Yamamoto K, Ikeda U, Furuhashi K, Irokawa M, Nakayama T, Shimada K. The coagulation system is activated in idiopathic cardiomyopathy. J Am Coll Cardiol. 1995;25:1634–40. 7539015 10.1016/0735-1097(95)00049-a

[19] Burczak DR, Scott CG, Julakanti RR, Kara Balla A, Swain WH, Ismail K, et al. Persistence of left atrial thrombus in patients with hypertrophic cardiomyopathy and atrial fibrillation. J Interv Card Electrophysiol. 2024;67:731–8. 37715077 10.1007/s10840-023-01642-5

[20] Arbelo E, Protonotarios A, Gimeno JR, Arbustini E, Barriales-Villa R, Basso C, et al. 2023 ESC guidelines for the management of cardiomyopathies. Eur Heart J. 2023;44:3503–626. 37622657 10.1093/eurheartj/ehad194

[21] Ommen SR, Ho CY, Asif IM, Balaji S, Burke MA, Day SM, et al. 2024 AHA/ACC/AMSSM/HRS/PACES/SCMR guideline for the management of hypertrophic cardiomyopathy: a report of the American Heart Association/American College of Cardiology Joint Committee on Clinical Practice Guidelines. Circulation. 2024;149:e1239–311. 38718139 10.1161/CIR.0000000000001250

[22] Siontis KC, Geske JB, Ong K, Nishimura RA, Ommen SR, Gersh BJ. Atrial fibrillation in hypertrophic cardiomyopathy: prevalence, clinical correlations, and mortality in a large high-risk population. J Am Heart Assoc. 2014;3: e001002. 24965028 10.1161/JAHA.114.001002 PMC4309084

[23] Rowin EJ, Link MS, Maron MS, Maron BJ. Evolving contemporary management of atrial fibrillation in hypertrophic cardiomyopathy. Circulation. 2023;148:1797–811. 38011245 10.1161/CIRCULATIONAHA.123.065037

[24] Zegkos T, Efthimiadis GK, Parcharidou DG, Gossios TD, Giannakoulas G, Ntelios D, et al. Atrial fibrillation in hypertrophic cardiomyopathy: a turning point towards increased morbidity and mortality. Hellenic J Cardiol. 2017;58:331–9. 28219794 10.1016/j.hjc.2017.01.027

[25] Ito T, Noda T, Nochioka K, Shiroto T, Yamamoto N, Sato H, et al. Clinical impact of atrial fibrillation progression in patients with heart failure with preserved ejection fraction: a report from the CHART-2 study. Europace. 2024;26: euae218. 39150084 10.1093/europace/euae218 PMC11368130

[26] De With RR, Marcos EG, Dudink EA, Spronk HM, Crijns HJ, Rienstra M, et al. Atrial fibrillation progression risk factors and associated cardiovascular outcome in well-phenotyped patients: data from the AF-RISK study. Europace. 2020;22:352–60. 31865391 10.1093/europace/euz339

[27] de Vos CB, Pisters R, Nieuwlaat R, Prins MH, Tieleman RG, Coelen RJ, et al. Progression from paroxysmal to persistent atrial fibrillation clinical correlates and prognosis. J Am Coll Cardiol. 2010;55:725–31. 20170808 10.1016/j.jacc.2009.11.040

[28] Wong JA, Conen D, Van Gelder IC, McIntyre WF, Crijns HJ, Wang J, et al. Progression of device-detected subclinical atrial fibrillation and the risk of heart failure. J Am Coll Cardiol. 2018;71:2603–11. 29880119 10.1016/j.jacc.2018.03.519

[29] Maron BJ, Haas TS, Maron MS, Lesser JR, Browning JA, Chan RH, et al. Left atrial remodeling in hypertrophic cardiomyopathy and susceptibility markers for atrial fibrillation identified by cardiovascular magnetic resonance. Am J Cardiol. 2014;113:1394–400. 24589281 10.1016/j.amjcard.2013.12.045

[30] Ho CY, López B, Coelho-Filho OR, Lakdawala NK, Cirino AL, Jarolim P, et al. Myocardial fibrosis as an early manifestation of hypertrophic cardiomyopathy. N Engl J Med. 2010;363:552–63. 20818890 10.1056/NEJMoa1002659 PMC3049917

[31] Guttmann OP, Pavlou M, O’Mahony C, Monserrat L, Anastasakis A, Rapezzi C, et al. Predictors of atrial fibrillation in hypertrophic cardiomyopathy. Heart. 2017;103:672–8. 27794017 10.1136/heartjnl-2016-309672

[32] Thomas L, Marwick TH, Popescu BA, Donal E, Badano LP. Left atrial structure and function, and left ventricular diastolic dysfunction: JACC state-of-the-art review. J Am Coll Cardiol. 2019;73:1961–77. 31000000 10.1016/j.jacc.2019.01.059

[33] Efremidis M, Bazoukis G, Vlachos K, Prappa E, Anastasakis A, Megarisiotou A, et al. Atrial substrate characterization in patients with atrial fibrillation and hypertrophic cardiomyopathy: evidence for an extensive fibrotic disease. J Electrocardiol. 2021;69:87–92. 34619440 10.1016/j.jelectrocard.2021.06.001

[34] Papavassiliu T, Germans T, Flüchter S, Doesch C, Suriyakamar A, Haghi D, et al. Cmr findings in patients with hypertrophic cardiomyopathy and atrial fibrillation. J Cardiovasc Magn Reson. 2009;11: 34. 19740409 10.1186/1532-429X-11-34 PMC2749835

[35] Pujadas S, Vidal-Perez R, Hidalgo A, Leta R, Carreras F, Barros A, et al. Correlation between myocardial fibrosis and the occurrence of atrial fibrillation in hypertrophic cardiomyopathy: a cardiac magnetic resonance imaging study. Eur J Radiol. 2010;75:e88-91. 20079992 10.1016/j.ejrad.2009.12.012

[36] Sivalokanathan S, Zghaib T, Greenland GV, Vasquez N, Kudchadkar SM, Kontari E, et al. Hypertrophic cardiomyopathy patients with paroxysmal atrial fibrillation have a high burden of left atrial fibrosis by cardiac magnetic resonance imaging. JACC Clin Electrophysiol. 2019;5:364–75. 30898240 10.1016/j.jacep.2018.10.016

[37] Zahid S, Malik T, Peterson C, Tarabanis C, Dai M, Katz M, et al. Conduction velocity is reduced in the posterior wall of hypertrophic cardiomyopathy patients with normal bipolar voltage undergoing ablation for paroxysmal atrial fibrillation. J Interv Card Electrophysiol. 2024;67:203–10. 36952090 10.1007/s10840-023-01533-9

[38] Qi D, Guan X, Liu X, Liu L, Liu Z, Zhang J. Slow conduction velocity predicts atrial fibrillation recurrence after radiofrequency ablation. J Cardiovasc Electrophysiol. 2024;35:461–8. 38282308 10.1111/jce.16193

[39] Santini L, Coppini R, Cerbai E. Ion channel impairment and myofilament Ca2+ sensitization: two parallel mechanisms underlying arrhythmogenesis in hypertrophic cardiomyopathy. Cells. 2021;10:2789. 34685769 10.3390/cells10102789 PMC8534456

[40] Autore C, Bernabò P, Barillà CS, Bruzzi P, Spirito P. The prognostic importance of left ventricular outflow obstruction in hypertrophic cardiomyopathy varies in relation to the severity of symptoms. J Am Coll Cardiol. 2005;45:1076–80. 15808767 10.1016/j.jacc.2004.12.067

[41] Maron MS, Olivotto I, Zenovich AG, Link MS, Pandian NG, Kuvin JT, et al. Hypertrophic cardiomyopathy is predominantly a disease of left ventricular outflow tract obstruction. Circulation. 2006;114:2232–9. 17088454 10.1161/CIRCULATIONAHA.106.644682

[42] Williams LK, Chan RH, Carasso S, Durand M, Misurka J, Crean AM, et al. Effect of left ventricular outflow tract obstruction on left atrial mechanics in hypertrophic cardiomyopathy. Biomed Res Int. 2015;2015: 481245. 26788503 10.1155/2015/481245 PMC4695661

[43] Kim DY, Seo J, Cho I, Hong GR, Ha JW, Shim CY. Prognostic implication of mitral valve disease and its progression in east asian patients with hypertrophic cardiomyopathy. J Am Heart Assoc. 2023;12: e024792. 36688372 10.1161/JAHA.121.024792 PMC9973656

[44] Tesic M, Travica L, Giga V, Jovanovic I, Trifunovic Zamaklar D, Popovic D, et al. Prognostic value of mitral regurgitation in patients with primary hypertrophic cardiomyopathy. Medicina (Kaunas). 2023;59:1798. 37893516 10.3390/medicina59101798 PMC10608691

[45] Yu EH, Omran AS, Wigle ED, Williams WG, Siu SC, Rakowski H. Mitral regurgitation in hypertrophic obstructive cardiomyopathy: relationship to obstruction and relief with myectomy. J Am Coll Cardiol. 2000;36:2219–25. 11127464 10.1016/s0735-1097(00)01019-6

[46] Zegkos T, Kamperidis V, Gossios T, Ntelios D, Parcharidou D, Papanastasiou CA, et al. Mitral regurgitation impact on left atrial myopathy in hypertrophic cardiomyopathy. Echocardiography. 2022;39:819–26. 35644869 10.1111/echo.15370

[47] Sciagrà R, Sotgia B, Olivotto I, Cecchi F, Nistri S, Camici PG, et al. Relationship between atrial fibrillation and blunted hyperemic myocardial blood flow in patients with hypertrophic cardiomyopathy. J Nucl Cardiol. 2009;16:92–6. 19152133 10.1007/s12350-008-9005-5

[48] Cecchi F, Sgalambro A, Baldi M, Sotgia B, Antoniucci D, Camici PG, et al. Microvascular dysfunction, myocardial ischemia, and progression to heart failure in patients with hypertrophic cardiomyopathy. J Cardiovasc Transl Res. 2009;2:452–61. 20560003 10.1007/s12265-009-9142-5

[49] Kim GH, Kim CM, Jang BH, Lee HH, Hong S, Eum SH, et al. Findings of cardiac magnetic resonance imaging in hypertrophic cardiomyopathy after 16 years. J Cardiovasc Ultrasound. 2016;24:239–42. 27721955 10.4250/jcu.2016.24.3.239 PMC5050313

[50] Wijffels MC, Kirchhof CJ, Dorland R, Allessie MA. Atrial fibrillation begets atrial fibrillation: a study in awake chronically instrumented goats. Circulation. 1995;92:1954–68. 7671380 10.1161/01.cir.92.7.1954

[51] Yakar Tuluce S, Kayikcioglu M, Tuluce K, Yilmaz MG, Ozdogan O, Aydın M, et al. Assessment of left atrial appendage function during sinus rhythm in patients with hypertrophic cardiomyopathy: transesophageal echocardiography and tissue doppler study. J Am Soc Echocardiogr. 2010;23:1207–16. 20869845 10.1016/j.echo.2010.08.016

[52] Seidman CE, Seidman JG. Identifying sarcomere gene mutations in hypertrophic cardiomyopathy: a personal history. Circ Res. 2011;108:743–50. 21415408 10.1161/CIRCRESAHA.110.223834 PMC3072749

[53] Bongini C, Ferrantini C, Girolami F, Coppini R, Arretini A, Targetti M, et al. Impact of genotype on the occurrence of atrial fibrillation in patients with hypertrophic cardiomyopathy. Am J Cardiol. 2016;117:1151–9. 26869393 10.1016/j.amjcard.2015.12.058

[54] Lee SP, Ashley EA, Homburger J, Caleshu C, Green EM, Jacoby D, et al. Incident atrial fibrillation is associated with MYH7 sarcomeric gene variation in hypertrophic cardiomyopathy. Circ Heart Fail. 2018;11: e005191. 30354366 10.1161/CIRCHEARTFAILURE.118.005191

[55] Zhang S, Wilson J, Madani M, Feld G, Greenberg B. Atrial arrhythmias and extensive left atrial fibrosis as the initial presentation of MYH7 gene mutation. JACC Clin Electrophysiol. 2018;4:1488–90. 30466861 10.1016/j.jacep.2018.07.016

[56] Milan DJ, Lubitz SA, Kääb S, Ellinor PT. Genome-wide association studies in cardiac electrophysiology: recent discoveries and implications for clinical practice. Heart Rhythm. 2010;7:1141–8. 20423731 10.1016/j.hrthm.2010.04.021 PMC2910183

[57] Guttmann OP, Pavlou M, O’Mahony C, Monserrat L, Anastasakis A, Rapezzi C, et al. Prediction of thrombo-embolic risk in patients with hypertrophic cardiomyopathy (HCM Risk-CVA). Eur J Heart Fail. 2015;17:837–45. 26183688 10.1002/ejhf.316 PMC4737264

[58] Tsuda T, Hayashi K, Fujino N, Konno T, Tada H, Nomura A, et al. Effect of hypertrophic cardiomyopathy on the prediction of thromboembolism in patients with nonvalvular atrial fibrillation. Heart Rhythm. 2019;16:829–37. 30503962 10.1016/j.hrthm.2018.11.029

[59] Dimitrow PP, Undas A, Bober M, Tracz W, Dubiel JS. Plasma biomarkers of endothelial dysfunction in patients with hypertrophic cardiomyopathy. Pharmacol Rep. 2007;59:715–20. 18195461

[60] Dimitrow PP, Undas A, Bober M, Tracz W, Dubiel JS. Obstructive hypertrophic cardiomyopathy is associated with enhanced thrombin generation and platelet activation. Heart. 2008;94: e21. 17923461 10.1136/hrt.2007.126896

[61] Debonnaire P, Joyce E, Hiemstra Y, Mertens BJ, Atsma DE, Schalij MJ, et al. Left atrial size and function in hypertrophic cardiomyopathy patients and risk of new-onset atrial fibrillation. Circ Arrhythm Electrophysiol. 2017;10: e004052. 28183843 10.1161/CIRCEP.116.004052

[62] Elliott PM, Anastasakis A, Borger MA, Borggrefe M, Cecchi F, Charron P, et al. 2014 ESC guidelines on diagnosis and management of hypertrophic cardiomyopathy: the task force for the diagnosis and management of hypertrophic cardiomyopathy of the European Society of Cardiology (ESC). Eur Heart J. 2014;35:2733–79. 25173338 10.1093/eurheartj/ehu284

[63] Tani T, Tanabe K, Ono M, Yamaguchi K, Okada M, Sumida T, et al. Left atrial volume and the risk of paroxysmal atrial fibrillation in patients with hypertrophic cardiomyopathy. J Am Soc Echocardiogr. 2004;17:644–8. 15163936 10.1016/j.echo.2004.02.010

[64] Raphael CE, Liew AC, Mitchell F, Kanaganayagam GS, Di Pietro E, Newsome S, et al. Predictors and mechanisms of atrial fibrillation in patients with hypertrophic cardiomyopathy. Am J Cardiol. 2020;136:140–8. 32950468 10.1016/j.amjcard.2020.09.006

[65] Carrick RT, Maron MS, Adler A, Wessler B, Hoss S, Chan RH, et al. Development and validation of a clinical predictive model for identifying hypertrophic cardiomyopathy patients at risk for atrial fibrillation: the HCM-AF score. Circ Arrhythm Electrophysiol. 2021;14: e009796. 34129346 10.1161/CIRCEP.120.009796

[66] Haruki S, Minami Y, Hagiwara N. Stroke and embolic events in hypertrophic cardiomyopathy: risk stratification in patients without atrial fibrillation. Stroke. 2016;47:936–42. 26941260 10.1161/STROKEAHA.115.012130

[67] Choi YJ, Kim B, Rhee TM, Lee HJ, Lee H, Park JB, et al. Augmented risk of ischemic stroke in hypertrophic cardiomyopathy patients without documented atrial fibrillation. Sci Rep. 2022;12:15785. 36138115 10.1038/s41598-022-19895-x PMC9499955

[68] Rowin EJ, Maron BJ, Haas TS, Garberich RF, Wang W, Link MS, et al. Hypertrophic Cardiomyopathy with left ventricular apical aneurysm: implications for risk stratification and management. J Am Coll Cardiol. 2017;69:761–73. 28209216 10.1016/j.jacc.2016.11.063

[69] Maron MS, Finley JJ, Bos JM, Hauser TH, Manning WJ, Haas TS, et al. Prevalence, clinical significance, and natural history of left ventricular apical aneurysms in hypertrophic cardiomyopathy. Circulation. 2008;118:1541–9. 18809796 10.1161/CIRCULATIONAHA.108.781401

[70] Mutlu B, Bayrak F, Kahveci G, Degertekin M, Eroglu E, Basaran Y. Usefulness of N-terminal pro-B-type natriuretic peptide to predict clinical course in patients with hypertrophic cardiomyopathy. Am J Cardiol. 2006;98:1504–6. 17126659 10.1016/j.amjcard.2006.06.053

[71] Kehl DW, Buttan A, Siegel RJ, Rader F. Clinical utility of natriuretic peptides and troponins in hypertrophic cardiomyopathy. Int J Cardiol. 2016;218:252–8. 27236124 10.1016/j.ijcard.2016.05.031

[72] D’Amato R, Tomberli B, Castelli G, Spoladore R, Girolami F, Fornaro A, et al. Prognostic value of N-terminal pro-brain natriuretic Peptide in outpatients with hypertrophic cardiomyopathy. Am J Cardiol. 2013;112:1190–6. 23871673 10.1016/j.amjcard.2013.06.018

[73] Kubo T, Kitaoka H, Okawa M, Yamanaka S, Hirota T, Baba Y, et al. Combined measurements of cardiac troponin I and brain natriuretic peptide are useful for predicting adverse outcomes in hypertrophic cardiomyopathy. Circ J. 2011;75:919–26. 21304210 10.1253/circj.cj-10-0782

[74] Moreno V, Hernández-Romero D, Vilchez JA, García-Honrubia A, Cambronero F, Casas T, et al. Serum levels of high-sensitivity troponin T: a novel marker for cardiac remodeling in hypertrophic cardiomyopathy. J Card Fail. 2010;16:950–6. 21111984 10.1016/j.cardfail.2010.07.245

[75] Kubo T, Kitaoka H, Yamanaka S, Hirota T, Baba Y, Hayashi K, et al. Significance of high-sensitivity cardiac troponin T in hypertrophic cardiomyopathy. J Am Coll Cardiol. 2013;62:1252–9. 23623916 10.1016/j.jacc.2013.03.055

[76] Kim K, Lee SD, Lee HJ, Kim H, Kim HR, Cho YH, et al. Role and clinical importance of progressive changes in echocardiographic parameters in predicting outcomes in patients with hypertrophic cardiomyopathy. J Cardiovasc Imaging. 2023;31:85–95. 37096673 10.4250/jcvi.2022.0053 PMC10133807

[77] Vasquez N, Ostrander BT, Lu DY, Ventoulis I, Haileselassie B, Goyal S, et al. Low left atrial strain is associated with adverse outcomes in hypertrophic cardiomyopathy patients. J Am Soc Echocardiogr. 2019;32:593–603. 30904367 10.1016/j.echo.2019.01.007

[78] Hussain K, Nso N, Tsourdinis G, Haider S, Mian R, Sanagala T, et al. A systematic review and meta-analysis of left atrial strain in hypertrophic cardiomyopathy and its prognostic utility. Curr Probl Cardiol. 2024;49: 102146. 37863460 10.1016/j.cpcardiol.2023.102146

[79] Lee HJ, Kim HK, Rhee TM, Choi YJ, Hwang IC, Yoon YE, et al. Left atrial reservoir strain-based left ventricular diastolic function grading and incident heart failure in hypertrophic cardiomyopathy. Circ Cardiovasc Imaging. 2022;15: e013556. 35439039 10.1161/CIRCIMAGING.121.013556

[80] Arnăutu SF, Morariu VI, Arnăutu DA, Tomescu MC, Dan TF, Jianu DC. Left atrial strain helps identifying the cardioembolic risk in transient ischemic attacks patients with silent paroxysmal atrial fibrillation. Ther Clin Risk Manag. 2022;18:213–22. 35299625 10.2147/TCRM.S359490 PMC8922319

[81] Abdelhamid S, Biomy R, Kabil H, Raslan M, Mostafa S. Association of left atrial deformation analysis by speckle tracking echocardiography with left atrial appendage thrombus in patients with primary valvular heart disease. Cureus. 2023;15: e35151. 36811128 10.7759/cureus.35151 PMC9938794

[82] Hong SP, Choi JY, Kim KS. Free floating left atrial thrombus disappearing during echocardiography. J Cardiovasc Ultrasound. 2013;21:152–3. 24198924 10.4250/jcu.2013.21.3.152 PMC3816168

[83] Agmon Y, Khandheria BK, Gentile F, Seward JB. Echocardiographic assessment of the left atrial appendage. J Am Coll Cardiol. 1999;34:1867–77. 10588196 10.1016/s0735-1097(99)00472-6

[84] Goldman ME, Pearce LA, Hart RG, Zabalgoitia M, Asinger RW, Safford R, et al. Pathophysiologic correlates of thromboembolism in nonvalvular atrial fibrillation: I. Reduced flow velocity in the left atrial appendage (the stroke prevention in atrial fibrillation [SPAF-III] study). J Am Soc Echocardiogr. 1999;12:1080–7. 10588784 10.1016/s0894-7317(99)70105-7

[85] Higuchi K, Akkaya M, Akoum N, Marrouche NF. Cardiac MRI assessment of atrial fibrosis in atrial fibrillation: implications for diagnosis and therapy. Heart. 2014;100:590–6. 23619986 10.1136/heartjnl-2013-303884

[86] Flett AS, Hasleton J, Cook C, Hausenloy D, Quarta G, Ariti C, et al. Evaluation of techniques for the quantification of myocardial scar of differing etiology using cardiac magnetic resonance. JACC Cardiovasc Imaging. 2011;4:150–6. 21329899 10.1016/j.jcmg.2010.11.015

[87] Maceira AM, Cosín-Sales J, Roughton M, Prasad SK, Pennell DJ. Reference left atrial dimensions and volumes by steady state free precession cardiovascular magnetic resonance. J Cardiovasc Magn Reson. 2010;12: 65. 21070636 10.1186/1532-429X-12-65 PMC2994941

[88] Tian D, Zhang J, He Y, Xiong Z, Zhao M, Hu S, et al. Predictive value of left atrial strain analysis in adverse clinical events in patients with hypertrophic cardiomyopathy: a CMR study. BMC Cardiovasc Disord. 2023;23:42. 36690952 10.1186/s12872-023-03069-2 PMC9869521

[89] Nagueh SF, Phelan D, Abraham T, Armour A, Desai MY, Dragulescu A, et al. Recommendations for multimodality cardiovascular imaging of patients with hypertrophic cardiomyopathy: an update from the American Society of Echocardiography, in collaboration with the American Society of Nuclear Cardiology, the Society for Cardiovascular Magnetic Resonance, and the Society of Cardiovascular Computed Tomography. J Am Soc Echocardiogr. 2022;35:533–69. 35659037 10.1016/j.echo.2022.03.012

[90] Spagnolo P, Giglio M, Di Marco D, Cannaò PM, Agricola E, Della Bella PE, et al. Diagnosis of left atrial appendage thrombus in patients with atrial fibrillation: delayed contrast-enhanced cardiac CT. Eur Radiol. 2021;31(3):1236–44. 32886202 10.1007/s00330-020-07172-2 PMC7880950

[91] Rajiah P, Alkhouli M, Thaden J, Foley T, Williamson E, Ranganath P. Pre- and postprocedural CT of transcatheter left atrial appendage closure devices. Radiographics. 2021;41:680–98. 33939541 10.1148/rg.2021200136

[92] Kim IC, Chang HJ, Cho IJ, Shim CY, Hong GR, Heo JH, et al. Benefit of four-dimensional computed tomography derived ejection fraction of the left atrial appendage to predict thromboembolic risk in the patients with valvular heart disease. Korean Circ J. 2019;49:173–80. 30468031 10.4070/kcj.2018.0152 PMC6351282

[93] Hosokawa T, Kawakami H, Tanabe Y, Fukuyama N, Yoshida K, Ohara K, et al. Left atrial strain assessment using cardiac computed tomography in patients with hypertrophic cardiomyopathy. Jpn J Radiol. 2023;41:843–53. 36811719 10.1007/s11604-023-01401-6 PMC10366261

[94] Kitaoka H, Carroll R, Eugene N, Teixeira BC, Matsuo Y, Kubo T. Oral anticoagulation in patients with hypertrophic cardiomyopathy and non-valvular atrial fibrillation in Japan. ESC Heart Fail. 2025;12:326–37. 39300752 10.1002/ehf2.15039 PMC11769629

[95] Noseworthy PA, Yao X, Shah ND, Gersh BJ. Stroke and bleeding risks in NOAC- and warfarin-treated patients with hypertrophic cardiomyopathy and atrial fibrillation. J Am Coll Cardiol. 2016;67:3020–1. 27339501 10.1016/j.jacc.2016.04.026

[96] Creta A, Hunter RJ, Earley MJ, Finlay M, Dhinoja M, Sporton S, et al. Non-vitamin K oral anticoagulants in hypertrophic cardiomyopathy patients undergoing catheter ablation of atrial fibrillation. J Cardiovasc Electrophysiol. 2020;31:2626–31. 32639666 10.1111/jce.14659

[97] Lee HJ, Kim HK, Jung JH, Han KD, Lee H, Park JB, et al. Novel oral anticoagulants for primary stroke prevention in hypertrophic cardiomyopathy patients with atrial fibrillation. Stroke. 2019;50:2582–6. 31340730 10.1161/STROKEAHA.119.026048

[98] Wu SJ, Chen YY, Chien YS, Kuo MJ, Li CH, Weng CJ, et al. Rhythm control better prevents stroke than rate control in patients with concomitant hypertrophic cardiomyopathy and atrial fibrillation: a nationwide population based cohort study with long-term follow-up. Acta Cardiol Sin. 2025;41:72–81. 39776924 10.6515/ACS.202501_41(1).20241111A PMC11701490

[99] Tops LF, Delgado V, Bertini M, Marsan NA, Den Uijl DW, Trines SA, et al. Left atrial strain predicts reverse remodeling after catheter ablation for atrial fibrillation. J Am Coll Cardiol. 2011;57:324–31. 21232671 10.1016/j.jacc.2010.05.063

[100] McKenna WJ, Harris L, Rowland E, Kleinebenne A, Krikler DM, Oakley CM, et al. Amiodarone for long-term management of patients with hypertrophic cardiomyopathy. Am J Cardiol. 1984;54:802–10. 6541429 10.1016/s0002-9149(84)80212-x

[101] Robinson K, Frenneaux MP, Stockins B, Karatasakis G, Poloniecki JD, McKenna WJ. Atrial fibrillation in hypertrophic cardiomyopathy: a longitudinal study. J Am Coll Cardiol. 1990;15:1279–85. 2329232 10.1016/s0735-1097(10)80014-2

[102] Harris L, McKenna WJ, Rowland E, Holt DW, Storey GC, Krikler DM. Side effects of long-term amiodarone therapy. Circulation. 1983;67:45–51. 6291807 10.1161/01.cir.67.1.45

[103] Tendera M, Wycisk A, Schneeweiss A, Poloński L, Wodniecki J. Effect of sotalol on arrhythmias and exercise tolerance in patients with hypertrophic cardiomyopathy. Cardiology. 1993;82:335–42. 8374931 10.1159/000175883

[104] Miller CA, Maron MS, Estes NA, Price LL, Rowin EJ, Maron BJ, et al. Safety, side effects and relative efficacy of medications for rhythm control of atrial fibrillation in hypertrophic cardiomyopathy. Am J Cardiol. 2019;123:1859–62. 30922542 10.1016/j.amjcard.2019.02.051

[105] Massera D, Sherrid MV, Adlestein E, Bokhari N, Alvarez IC, Wu WY, et al. Disopyramide revisited for treatment of symptomatic obstructive hypertrophic cardiomyopathy: efficacy and safety in patients treated for at least 5 years. J Am Heart Assoc. 2025;14: e037639. 39817530 10.1161/JAHA.124.037639 PMC12054425

[106] Sherrid MV, Barac I, McKenna WJ, Elliott PM, Dickie S, Chojnowska L, et al. Multicenter study of the efficacy and safety of disopyramide in obstructive hypertrophic cardiomyopathy. J Am Coll Cardiol. 2005;45:1251–8. 15837258 10.1016/j.jacc.2005.01.012

[107] Echt DS, Liebson PR, Mitchell LB, Peters RW, Obias-Manno D, Barker AH, et al. Mortality and morbidity in patients receiving encainide, flecainide, or placebo. The cardiac arrhythmia suppression trial. N Engl J Med. 1991;324:781–8. 1900101 10.1056/NEJM199103213241201

[108] Faraz F, Rehman MEU, Sabir B, Ghaffar A, Iftikhar A, Maqsood A, et al. Efficacy of catheter ablation for atrial fibrillation in hypertrophic cardiomyopathy: a systematic review and meta-analysis. Curr Probl Cardiol. 2023;48: 101524. 36455792 10.1016/j.cpcardiol.2022.101524

[109] Dinshaw L, Münkler P, Schäffer B, Klatt N, Jungen C, Dickow J, et al. Ablation of atrial fibrillation in patients with hypertrophic cardiomyopathy: treatment strategy, characteristics of consecutive atrial tachycardia and long-term outcome. J Am Heart Assoc. 2021;10: e017451. 33455428 10.1161/JAHA.120.017451 PMC7955439

[110] Zheng S, Jiang W, Dai J, Li K, Shi H, Wu W, et al. Five-year outcomes after catheter ablation for atrial fibrillation in patients with hypertrophic cardiomyopathy. J Cardiovasc Electrophysiol. 2020;31:621–8. 31930545 10.1111/jce.14349

[111] Lee JH, Cho I, Choi SH, Yu HT, Kim TH, Uhm JS, et al. Clinical and pathophysiologic determinants of catheter ablation outcome in hypertrophic cardiomyopathy with atrial fibrillation. J Arrhythm. 2024;40:479–88. 38939784 10.1002/joa3.13061 PMC11199821

[112] Contreras-Valdes FM, Buxton AE, Josephson ME, Anter E. Atrial fibrillation ablation in patients with hypertrophic cardiomyopathy: long-term outcomes and clinical predictors. J Am Coll Cardiol. 2015;65:1485–7. 25857916 10.1016/j.jacc.2014.12.063

[113] Ezzeddine FM, Agboola KM, Hassett LC, Killu AM, Del-Carpio Munoz F, DeSimone CV, et al. Catheter ablation of atrial fibrillation in patients with and without hypertrophic cardiomyopathy: systematic review and meta-analysis. Europace. 2023;25: euad256. 37595138 10.1093/europace/euad256 PMC10498139

[114] Agarwal S, Munir MB, Patel H, DeSimone CV, Deshmukh A, Asad ZU. Outcomes of catheter ablation for atrial fibrillation in patients with hypertrophic cardiomyopathy. Am J Cardiol. 2023;207:1–3. 37717284 10.1016/j.amjcard.2023.08.156

[115] Kharbanda RK, Ramdat Misier NL, Van den Eynde J, El Mathari S, Tomšič A, Palmen M, et al. Outcomes of concomitant surgical ablation in patients undergoing surgical myectomy for hypertrophic obstructive cardiomyopathy: a systematic review and meta-analysis. Int J Cardiol. 2023;387: 131099. 37263356 10.1016/j.ijcard.2023.05.049

[116] Gribissa S, Kueffer T, Knecht S, Waintraub X, Badenco N, Charron P, et al. Pulsed-field vs thermal catheter ablation of atrial fibrillation in patients with hypertrophic cardiomyopathy. JACC Clin Electrophysiol. 2025;11:1205–15. 40088215 10.1016/j.jacep.2025.01.009

[117] Agarwal S, Munir MB, DeSimone CV, Deshmukh A, Alkhouli MA, Asad ZU. Outcomes of patients with hypertrophic cardiomyopathy undergoing percutaneous left atrial appendage occlusion. J Interv Card Electrophysiol. 2024;67:851–4. 38411856 10.1007/s10840-024-01772-4

[118] Aglan A, Fath AR, Maron BJ, Maron MS, Prasad A, Almomani A, et al. Percutaneous left atrial appendage closure for stroke prevention in hypertrophic cardiomyopathy patients with atrial fibrillation. Heart Rhythm. 2024;21:1677–83. 38797308 10.1016/j.hrthm.2024.05.038

[119] Khawaja T, Majmundar M, Zuzek Z, Arora S, Attizzani GF, Filby SJ, et al. Surgical and transcatheter left atrial appendage closure in patients with atrial fibrillation and hypertrophic cardiomyopathy. Europace. 2023;25: euad101. 37143414 10.1093/europace/euad101 PMC10228613

[120] Glikson M, Wolff R, Hindricks G, Mandrola J, Camm AJ, Lip GY, et al. EHRA/EAPCI expert consensus statement on catheter-based left atrial appendage occlusion: an update. Europace. 2020;22:184. 31504441 10.1093/europace/euz258

[121] January CT, Wann LS, Calkins H, Chen LY, Cigarroa JE, Cleveland JC, et al. 2019 AHA/ACC/HRS focused update of the 2014 AHA/ACC/HRS guideline for the management of patients with atrial fibrillation: a report of the American College of Cardiology/American Heart Association Task Force on Clinical Practice Guidelines and the Heart Rhythm Society in collaboration with the Society of Thoracic Surgeons. Circulation. 2019;140:e125–51. 30686041 10.1161/CIR.0000000000000665

[122] Holmes DR, Reddy VY, Turi ZG, Doshi SK, Sievert H, Buchbinder M, et al. Percutaneous closure of the left atrial appendage versus warfarin therapy for prevention of stroke in patients with atrial fibrillation: a randomised non-inferiority trial. Lancet. 2009;374:534–42. 19683639 10.1016/S0140-6736(09)61343-X

[123] Holmes DR, Kar S, Price MJ, Whisenant B, Sievert H, Doshi SK, et al. Prospective randomized evaluation of the Watchman Left Atrial Appendage Closure device in patients with atrial fibrillation versus long-term warfarin therapy: the PREVAIL trial. J Am Coll Cardiol. 2014;64:1–12. 24998121 10.1016/j.jacc.2014.04.029

[124] Olivotto I, Oreziak A, Barriales-Villa R, Abraham TP, Masri A, Garcia-Pavia P, et al. Mavacamten for treatment of symptomatic obstructive hypertrophic cardiomyopathy (EXPLORER-HCM): a randomised, double-blind, placebo-controlled, phase 3 trial. Lancet. 2020;396:759–69. 32871100 10.1016/S0140-6736(20)31792-X

[125] Desai MY, Okushi Y, Wolski K, Geske JB, Owens A, Saberi S, et al. Mavacamten-associated temporal changes in left atrial function in obstructive HCM: insights from the VALOR-HCM trial. JACC Cardiovasc Imaging. 2025;18:251–62. 39254622 10.1016/j.jcmg.2024.08.005

[126] Wessly P, Lazzara GE, Buergler JM, Nagueh SF. Early observations on effects of mavacamten on left atrial function in obstructive hypertrophic cardiomyopathy patients. JACC Cardiovasc Imaging. 2023;16:1633–4. 37452821 10.1016/j.jcmg.2023.06.008

[127] Lu R, Lumish HS, Hasegawa K, Maurer MS, Reilly MP, Weiner SD, et al. Prediction of new-onset atrial fibrillation in patients with hypertrophic cardiomyopathy using machine learning. Eur J Heart Fail. 2025;27:275–84. 39694602 10.1002/ejhf.3546 PMC11859767

[128] Wang Z, Liao H, He S, Chen X. Performance and validation of R-CHA2DS2VASc score for thromboembolism in patients with hypertrophic cardiomyopathy. Hellenic J Cardiol. 2020;61:313–7. 31472238 10.1016/j.hjc.2019.08.001

